# Wt-1 Expression Linked to Nitric Oxide Availability during Neonatal Obstructive Nephropathy

**DOI:** 10.1155/2013/401750

**Published:** 2013-10-31

**Authors:** Luciana Mazzei, Walter Manucha

**Affiliations:** ^1^Área de Fisiopatología, Departamento de Patología, Facultad de Ciencias Médicas, Universidad Nacional de Cuyo, Centro Universitario, CP 5500, Mendoza, Argentina; ^2^National Council of Scientific and Technical Research of Argentina (IMBECU-CONICET), CP 5500, Mendoza, Argentina

## Abstract

The *wt-1* gene encodes a zinc finger DNA-binding protein that acts as a transcriptional activator or repressor depending on the cellular or chromosomal context. The *wt-1* regulates the expression of a large number of genes that have a critical role in kidney development. Congenital obstructive nephropathy disrupts normal renal development and causes chronic progressive interstitial fibrosis, which contributes to renal growth arrest, ultimately leading to chronic renal failure. Wt-1 is downregulated during congenital obstructive nephropathy, leading to apoptosis. Of great interest, nitric oxide bioavailability associated with heat shock protein 70 (Hsp70) interaction may modulate *wt-1* mRNA expression, preventing obstruction-induced cell death during neonatal unilateral ureteral obstruction. Moreover, recent genetic researches have allowed characterization of many of the complex interactions among the individual components cited, but the realization of new biochemical, molecular, and functional experiments as proposed in our and other research labs allows us to establish a deeper level of commitment among proteins involved and the potential pathogenic consequences of their imbalance.

## 1. Wt-1 Expression and Isoforms

The *wt*-*1* gene encodes a zinc finger DNA-binding protein that acts as a transcriptional activator or repressor depending on the cellular or chromosomal context. Wilms tumor locus was narrowed down to a region of less than 345 kb on human chromosome 11p13. The *wt*-*1* mRNA has three translation start sites resulting in three isoforms of the protein with different molecular weights: 62–64 kDa, 52–54 kDa, and 36–38 kDa. Typical protein WT-1 is 52–54 kDa isoform [1]. In addition, it has 4 major isoforms, due to the insertion of 3 amino acids (KTS) between zinc fingers 3 and 4 and the insertion of an alternatively spliced 17-amino acid segment encoded by exon 5 in the middle of the protein [2]. Florio et al. stated that at least 24 different WT-1 isoforms are produced by alternative splicing and the use of alternate translation initiation sites [3]. Previously, Scharnhorst et al. described additional WT-1 isoforms with distinct transcription-regulatory properties, indicating further the complexity of WT-1 expression and activity. They stated that 32 WT-1 protein forms had been described [4]. 

The 429-amino acid polypeptide had features suggesting a role in transcriptional regulation: the presence of 4 zinc finger domains and a region rich in proline and glutamine. The conservation in structure and relative levels of the 4 *wt-1* mRNA species suggests that each encoded polypeptide makes a significant contribution to normal gene function. The control of cellular proliferation and differentiation exerted by the *wt-1* gene products may involve interactions between the 4 polypeptides with distinct targets and functions [5]. Its activity is controlled through phosphorylation by protein kinase A (PKA). PKA-dependent WT-1 phosphorylation (originally shown by Ye et al. [6]) results in translocation of WT-1 from the nucleus to the cytosol, a process that interferes with WT-1 transcriptional activities [7].

## 2. WT-1 Functions on the Kidney Development

WT-1 is required for normal formation of the genitourinary system and mesothelial tissues. Wilms tumor gene was expressed specifically in the condensed mesenchyme, renal vesicle, and glomerular epithelium of the developing kidney; in the related mesonephric glomeruli; and in cells approximating these structures in tumors [8]. One of the more significant results in the field of renal development was the finding that knocking out the *wt*-*1* gene in mice results in anephric animals [9]. The other main sites of expression were the genital ridge, fetal gonad, and mesothelium [8]. This nuclear protein may be important in the maintenance of ovarian follicles at early stages of development [10].

*Wt*-*1* is likely to be a master control gene that regulates the expression of a large number of genes that have a critical role in kidney development [11]. Due to its roles in development and cell proliferation, polymorphisms within the *wt*-*1* gene can result in malignancies such as leukemia and Wilms tumor [7]. Wilms' tumor 1 gene, *wt*-*1*, is homozygously mutated in a subset of Wilms' tumors. Heterozygous mutations in *wt*-*1* give rise to congenital anomalies [4]. Both constitutional and somatic mutations disrupting the DNA-binding domain of WT-1 result in a potentially dominant-negative phenotype. In generating inducible cell lines expressing wild-type isoforms of WT-1 as well as WT-1 mutants, Englert et al. observed dramatic differences in the subnuclear localization of the induced proteins. The WT-1 isoform that binds with high affinity to a defined DNA target, WT-1(−KTS), was diffusely localized throughout the nucleus. In contrast, expression of an alternative splicing variant with reduced DNA-binding affinity, WT-1(+KTS), or WT-1 mutants with a disrupted zinc finger domain resulted in a speckled pattern of expression within the nucleus [12]. 

Localization to subnuclear clusters required the N terminus of WT-1 and coexpression of a truncated WT-1 mutant and wild-type WT-1(−KTS) resulted in a physical association, the redistribution of WT-1(−KTS) from a diffuse to a speckled pattern, and the inhibition of its transactivational activity. These observations suggested to the authors that different WT-1 isoforms and WT-1 mutants have distinct subnuclear compartments [12]. Dominant-negative WT-1 proteins physically associate with wild-type WT-1 *in vivo* and may result in its sequestration within subnuclear structures [12].

## 3. Nitric Oxide Linked to WT-1 during Obstructive Nephropathy

Changes in *wt*-*1* expression pattern during ontogenesis suggest a significant role both during embryonic as well as during fetal and postnatal, urogenital development. Moreover, gene targeting studies have shown that Wt-1 is absolutely necessary for urogenital formation at different developmental stages [9]. Wilms tumor gene, identified as missing or mutated in embryonic kidney cancer cells [13], is a versatile gene that controls transitions between the mesenchymal and epithelial state of cells in a tissue-context dependent manner [14].

It is downregulated during congenital obstructive nephropathy, leading to apoptosis [15]. Congenital obstruction is the primary cause of end-stage renal disease in children. The kidney shows profound morphologic and functional changes. The physiologic developmental kidney program is disturbed in the most advanced cases, arguing for altered temporal/spatial expression of genes which control normal nephrogenesis [16]. However, the mechanisms underlying chronic obstructive nephropathy have not yet been completely elucidated. Relating changes in gene expression to phenotypic patterns in human congenital obstructive nephropathy represent an advance in the identification of the genes/proteins that play important roles [17]. 

Changes in gene expression patterns during development and maturation of the kidney modulate a series of events that are responsible for extraordinary structural and functional complexity. Congenital obstructive nephropathy disrupts normal renal development and causes chronic progressive interstitial fibrosis, which contributes to renal growth arrest, ultimately leading to chronic renal failure. Therefore, renal growth and development are severely affected by obstructive injury through complex interactions among regulators of cell proliferation and apoptosis [15]. 

Major regulators of mesenchymal-epithelial transformation and collecting duct and tubular development such as WT-1 and Sall1 are decreased with obstruction. Excessive apoptosis is an undisputed mechanism in these processes, mediated by decreased expression of apoptosis inhibiting genes (*bcl2, hgf, igf, bmp7*) and overexpression of proapoptotic genes like *Bax *and *TGF-beta*. Renin and angiotensin type II receptor (AT_2_r) implicated in renal vascular development are decreased [16]. 

The stimuli responsible for the induction of apoptosis are varied and include mechanical stress secondary to pulsatile retrograde pressure transfer from ureteric peristalsis [18], hypoxia [19] occurring secondary to reductions in renal blood flow in the obstructed kidney, and inflammatory reactions [20] caused by the influx of innate immune cells in response to chemotactic signals from the damaged renal parenchyma. Associated oxidative stress is a phenomenon common to all the injurious stimuli described above and has been widely recognized by ourselves and others as a key contributor to renal injury following obstruction [21–23].

Experimentally induced unilateral ureteral obstruction (UUO) represents an interesting model for studying early fibrogenesis. This model mimics the different stages of human neonatal obstructive nephropathy leading to apoptosis, tubule-interstitial fibrosis, epithelial-mesenchymal transition, and tubular atrophy [20]. Further, it reflects important aspects of inflammation that are prominent in human kidney diseases contributing to the UUO pathophysiology [21, 24, 25], as well in chronic kidney disease (CKD) [26], revealing useful biomarkers of renal disease progression as well as new therapies, which are required to allow intervention before the establishment of irreversible renal injury [27]. 

Studies in neonatal rats may provide insight into the functional development of the kidney, since nephrogenesis continues at a rapid pace up to day 8 after birth and is virtually complete by days 14–19. In this regard, experimentally induced UUO has emerged as an interesting model for studying neonatal hydronephrosis and for the assessment of potential therapeutic approaches. This model mimics, in an accelerated manner, the different stages of human neonatal hydronephrosis leading to tubulointerstitial fibrosis, apoptosis, epithelial-mesenchymal transition, and tubular atrophy. Thus, UUO in neonatal rats impairs nephrogenesis, glomerular maturation, and tubular cellular proliferation [28].

While pharmacological protection from tubular apoptosis is clearly likely to be of benefit to the preservation of renal structure and function in the adult kidney, the situation in neonatal obstruction in rodents and in utero obstruction in humans is somewhat more nuanced. This is based on the fact that in these cases the kidney is still passing through the nephrogenic program, and in order for a pharmacological agent to be of true worth, it must not only be cytoprotective but also allow for preservation of nephrogenesis, in order that the developing kidney achieves its optimal quota of excretory units and hence its optimal function [23]. The younger the age at which UUO is performed, the more severe the growth impairment of the ipsilateral kidney [29]. Hence UUO results in altered cell proliferation and apoptosis in the neonatal rat kidney [15]. These processes also occur in the developing obstructed human kidney. They are mediated by complex signaling pathways, including the heat shock protein response associated with nitric oxide (NO) interaction [30, 31]. 

Johannesen et al. have shown functional interactions between the gene promoter of the main source of the inducible nitric oxide synthase (*iNOS*) and WT-1, where *iNOS* promoter is strain-dependently regulated [32], which may relate to strain-dependent differences in WT-1 transcription factor expression and a modulatory role of NO in the proliferation of T cells expressing WT-1 has been suggested [7]. Interestingly, a cytoprotective role of NO associated with heat shock protein 70 (*Hsp70)* expression in neonatal hydronephrosis was recently shown [31, 33]. A 3-hydroxy-3-methylglutaryl-coenzyme-A reductase inhibitor, rosuvastatin, has a protective effect against podocyte apoptosis “*in vitro” *[34]. Renoprotection by statins has been recently associated with increased NO availability [31, 35, 36]. After neonatal UUO, rosuvastatin prevents apoptosis through an increase in NO bioavailability, which in turn is linked to higher *hsp70* expression [15, 36].

Moreover, rosuvastatin (hydrophilic statin) may have potential utility as a therapeutic option in renal diseases that are characterized by inflammation and fibrosis, independently of changes in blood pressure and plasma lipid levels [37]. Randomized studies with HMG-CoA reductase inhibitors (statins) have shown that their major adverse effects are associated with muscle and liver toxicity. However, rosuvastatin seems safe in this regard [38]. In addition, we found no significant changes in either body mass or kidney mass in rosuvastatin-treated animals [15].

Many signals may positively or negatively affect the rat kidney after UUO by altering regulatory proteins that initiate apoptosis and inducing changes in mitochondrial function [20, 39]. NO is able to either induce or inhibit apoptosis in different circumstances [40], while factors such as *bcl*_*2*_ have an antiapoptotic effect [15].

Decreased NO and *iNOS/hsp70* expressions associated with *wt-1* low expression were shown in obstructed kidneys [41]. Apoptosis was induced and it was associated with an increased *bax/bcl*_*2*_ ratio.

Conversely*, iNOS/hsp70* upregulation and an increased *wt*-*1* mRNA expression, without an apoptotic response, were observed in the cortex of obstructed kidneys of rosuvastatin-treated rats. NO also modulated *hsp70* and *wt*-*1* mRNA expression in Madin-Darby canine kidney (MDCK) cells. “*In vivo” *experiments with NO modulators result in *wt*-*1* mRNA expression associated with NO level [15]. Rosuvastatin may modulate *wt*-*1* mRNA expression through renal NO bioavailability, preventing neonatal obstruction-induced apoptosis associated with Hsp70 interaction [41].

Glomerular development fundamentally relies on the correct maturation of three cell lineages: the epithelial (parietal and visceral epithelia of Bowman's capsule), the endothelial (glomerular capillaries), and the mesenchymal (mesangial cells). The epithelial component forms the proximal end of the excretory arm of the renal tubule via reciprocal inductive events occurring between the ureteric bud and the metanephric mesenchyme. In order to epithelialize, the mesenchyme must receive and respond to bone morphogenetic protein 7 (BMP 7) signaling and also express key isoforms of Wilms' tumor-1 (WT-1) zinc-finger transcription factor. We have shown that rosuvastatin preserves both BMP-7 and WT-1 levels in neonatal UUO [23].

Obstructive nephropathy is also associated with downexpression of genes related to renal vascular development as renin and angiotensin II AT_2_ receptor [16]. Our group has shown that selective blockade of angiotensin II AT_1_ receptor decreases renal interstitial fibrosis in UUO, and this effect is associated with *iNOS* activity and expression [22] as well as with *hsp70* expression [42]. In addition, Hsp70 is involved in cellular signaling pathways related to apoptosis, protein folding, and membrane translocation and in modulating the activity of tumor suppressor proteins, including p53 and WT-1 [43]. 

It has been hypothesized that NO may participate in the maintenance of renal function and in the maturation of developing kidneys [44, 45].

Apoptosis is the principal mechanism that leads to tubular atrophy during the neonatal obstructive nephropathy process. Excessive cell death is mediated by decreased expression of apoptosis inhibiting genes like *bcl2* and overexpression of proapoptotic genes like *bax*. Correspondingly, 14 days of obstruction led to the induction of apoptosis regulated by mitochondrial signal pathway through a proapoptotic increased *bax/bcl2* ratio and, consequently, an increased activity of caspase 3 [33].

Therefore, it may be suggested that in certain cellular contexts, WT-1 exhibits antiapoptotic potential through the transcriptional upregulation of *bcl2* [46].

Future experiments may explore *wt*-*1* mRNA expression during UUO after release and its possible involvement in renal functional and structural recovery. Recently, Yoo et al. reported that, in complete UUO in mice, *iNOS* attenuates apoptosis and increases renal parenchymal thickness [47]. Other results corroborate previous reports in which NO has been proposed as a key factor modulating apoptosis in UUO [20, 33, 48, 49]. 

Many studies have shown the benefits of NO donors and deleterious effects of NO inhibitors in obstructive nephropathy. NO stimulates the expression of enzymes and transcription factors involved in DNA repair and modulation of apoptosis, such as the tumor suppressor p53. In turn, p53 interacts with WT-1 and modulates its ability to regulate the transcription of its respective target genes [50]. Consequently, it might be expected that increased NO availability induces *p53* and *wt*-*1* mRNA expression, as indeed shown by our results [15]. Moreover, WT-1 can stabilize p53, adjust its transactivational properties, and inhibit its ability to induce apoptosis without affecting cellular arrest [51]. This effect may explain the elevated p53 levels observed by other authors during obstructive nephropathy apoptosis induction [52–55]. 

NO can oxidize intracellular glutathione and modify cellular antioxidant levels. This stimulates heat shock protein induction as in Hsp32 and Hsp70. NO generated from several compounds induces Hsp70 expression in arterial smooth muscle cells [56]. Hsp70 plays an important role in nascent protein folding, reassembling denatured proteins and solubilized protein aggregates [57]. Moreover, it is involved in cellular signaling pathways linked to apoptosis and membrane translocation and in the modulation of the activities of tumor suppressor proteins, including p53 and WT-1 [58]. Inducible Hsp70 expression has been shown to enhance the survival of mammalian cells exposed to numerous types of stimuli that induce stress and apoptosis [59]. Furthermore, inducible Hsp70 protects renal epithelial cells from apoptosis by caspase activation [60]. WT-1 and Hsp70 are physically associated in embryonic rat kidney cells, where the amino-terminal transactivation domain of WT-1 is required for binding to Hsp70, and domain expression itself is sufficient to induce Hsp70 expression [43]. There was a significant decrease in Hsp70 expression associated with low NO availability in neonatal obstructed cortex [15]. Interestingly, rosuvastatin treatment induced a significant increase in *iNOS* expression and nitrite levels, and this was related to enhance Hsp70 expression. Induction of Hsp70 not only protects cells from damage due to apoptosis induction but also from damage due to oxidative injury. Hence, NO can induce cytoprotection in early obstructed kidney cortex tubular epithelial cells through the stimulation of Hsp70 expression [33]. Previously, Hegarty et al. showed in a well characterized renal epithelial cell line (MDCK) under mechanical strain (hydronephrosis cellular model) an increased susceptibility to apoptosis [61]. The cellular effects of mechanical strain were reversed by sodium nitroprusside and L-arginine. Since about 80% of total kidney mass is composed of tubular epithelial cells, it seems reasonable to infer that “*in vivo” *results represent mostly phenomena affecting this cell population. To corroborate this, basal *wt*-*1* mRNA expression and the effects of NO availability were also studied “*in vitro” *in MDCK cells. Low NO availability was associated with low expression of Hsp70 and WT-1. 

NO donors did not significantly change the Hsp70 expression, nor did the NO precursor L-arginine induce any changes in *wt*-*1* mRNA expression. However, *wt*-*1* mRNA expression was increased when MDCK cells were incubated for 72 hours with NO donors. These results in MDCK cells suggest a greater susceptibility to low NO levels associated with low *wt*-*1* mRNA expression [15].

While it cannot be simply assumed that “*in vitro” *results faithfully reproduce “*in vivo” *mechanisms, in both cases nitric oxide levels associated with *hsp70* mRNA expression seem to modulate *wt*-*1* mRNA expression. This proposed mechanism is further supported by the results of treating neonatal rats with NO modulators, where a close relationship between endogenous nitrite levels and *wt*-*1/hsp70* mRNA expression was found. The p53 protein interacts with members of the Hsp70 chaperone family which can regulate its function [62, 63]. In this regard, neonatal UUO shows low p53 and Hsp70 expressions, which are increased in association with higher NO levels under rosuvastatin treatment. Conversely, MDCK cells with NO deprivation expressed low *hsp70* and *p53* mRNA levels. These observations suggest a potential role for NO bioavailability and Hsp70 interaction during kidney differentiation.

In conclusion, unilateral ureteral obstruction in neonatal rodents can be used as a paradigm for “*in uteri” *obstruction in humans and a platform for studying the potential of novel therapies for congenital obstructive nephropathy. Nitric oxide bioavailability associated with Hsp70 interaction may modulate *wt*-*1* mRNA expression, preventing obstruction-induced cell death during neonatal unilateral ureteral obstruction.
